# Triclosan changes community composition and selects for specific bacterial taxa in marine periphyton biofilms in low nanomolar concentrations

**DOI:** 10.1007/s10646-020-02246-9

**Published:** 2020-07-13

**Authors:** Eriksson Karl Martin, Sanli Kemal, Nilsson Rickard Henrik, Eiler Alexander, Corcoll Natalia, Johansson Carl Henrik, Backhaus Thomas, Blanck Hans, Kristiansson Erik

**Affiliations:** 1 Department of Mechanics and Maritime Sciences, Chalmers University of Technology, Gothenburg, Sweden; 2 Department of Biological and Environmental Sciences, University of Gothenburg, Gothenburg, Sweden; 3 Department of Ecology and Genetics, Uppsala University, Uppsala, Sweden; 4 Department of Mathematical Sciences, Chalmers University of Technology, Gothenburg, Sweden

**Keywords:** Amplicon sequencing, Metabarcoding, rRNA, Community structure, Antimicrobial compounds, e-DNA

## Abstract

The antibacterial agent Triclosan (TCS) is a ubiquitous environmental contaminant due to its widespread use. Sensitivity to TCS varies substantially among eu- and pro-karyotic species and its risk for the marine environment remains to be better elucidated. In particular, the effects that TCS causes on marine microbial communities are largely unknown. In this study we therefore used 16S amplicon rDNA sequencing to investigate TCS effects on the bacterial composition in marine periphyton communities that developed under long-term exposure to different TCS concentrations. Exposure to TCS resulted in clear changes in bacterial composition already at concentrations of 1 to 3.16 nM. We conclude that TCS affects the structure of the bacterial part of periphyton communities at concentrations that actually occur in the marine environment. Sensitive taxa, whose abundance decreased significantly with increasing TCS concentrations, include the *Rhodobiaceae* and *Rhodobacteraceae* families of *Alphaproteobacteria*, and unidentified members of the Candidate division *Parcubacteria*. Tolerant taxa, whose abundance increased significantly with higher TCS concentrations, include the families *Erythrobacteraceae* (*Alphaproteobacteria*), *Flavobacteriaceae* (*Bacteroidetes*)*, Bdellovibrionaceae* (*Deltaproteobacteria*), several families of *Gammaproteobacteria*, and members of the Candidate phylum *Gracilibacteria*. Our results demonstrate the variability of TCS sensitivity among bacteria, and that TCS can change marine bacterial composition at concentrations that have been detected in the marine environment.

## Introduction

Triclosan (TCS, 5-chloro-2-(2,4-dichloro-phenoxy)-phenol, CAS 3380-34-5) is an antibacterial agent commonly used in personal care products (PCP), household cleaning products, textiles, and plastics. The annual usage of TCS and has been estimated at 300 tons in USA in 2005 (Halden and Paull 2005) and 450 tons in Europe in 2010 (SCCS 2010). Approximately 85% of the TCS produced is used in PCPs (SCCS 2010), and the compound is therefore discharged continuously into the aquatic environment. TCS has become an ubiquitous pollutant, occurring in all environmental compartments (Bedoux et al. 2012). As reviewed by Bedoux et al. (2012), TCS concentrations of up to 0.024, 0.047 and 0.1 nM have been reported for coastal waters in Europe, USA, and China, respectively. Furthermore, 0.036 nM was detected in the coastal waters outside Singapore (Bayen et al. 2013), 0.55 nM was measured at the Swedish west coast (Remberger et al. 2002), and a concentration as high as 1.1 nM was detected in Cadiz Bay in Spain (Pintado-Herrera et al. 2014). Given this widespread occurrence, von der Ohe et al. (2012) identified the compound as a priority pollutant in freshwater ecosystems, and Maruya et al. (2015) labeled TCS a contaminant of emerging concern for the marine environment, based on sediment core data in which TCS concentrations increased from the early 1970s to 2007. In 2016, the European Commission decided to ban the use of triclosan in human hygiene biocidal products from 2017 (European Commission 2016), and some manufacturers have phased out the compound from some of their products globally (Halden et al. 2017).

The environmental risk of TCS has been assessed with conflicting results. A probabilistic risk assessment by Capdevielle et al. (2008) concluded that the risks from TCS at environmental concentrations were small, whereas several other studies indicated more clear environmental hazards and risks (Brausch and Rand 2011; Chalew and Halden 2009; Reiss et al. 2002; Wilson et al. 2003; von der Ohe et al. 2012). In a recent global assessment Guo and Iwata (2017) calculated risk quotients, i.e. ratios of measured environmental concentrations and predicted no effect concentrations, to be between 0.49–9.5 for surface waters. If such risk ratios are above 1, an unacceptable risk exists. It should be pointed out, that those assessments do not assess risks to the marine environment, due to a lack of adequate data, in particular for marine bacteria. In a recent paper, more than 200 scientists signed the so-called Florence statement on the hazards of from triclosan and triclocarban, and recommended that the use of these compounds should be avoided except for specific cases where they provide an evidence-based health benefit and there is adequate evidence demonstrating they are safe (Halden et al. 2017).

The mechanism of action of TCS in bacteria has been identified as the inhibition of type II fatty acid synthesis through binding to the enoyl-acyl carrier protein (enoyl-ACP) reductase (McMurry et al. 1998). Different bacterial species have different conformations of the TCS binding site in the enoyl-ACP reductase which affects the affinity to TCS and thereby TCS sensitivity (Pidugu et al. 2004). Johnson et al. (2009) also report a broad range of bacterial sensitivities to TCS, ranging from 100 nM to 300 µM. Although the inhibition of fatty acid synthesis is a well-described mechanism of action, Escalada et al. (2005) concluded that the toxicity of TCS to bacteria cannot be explained solely by this mechanism. Studies have also shown that TCS induces cell membrane destabilization (Villalaín et al. 2001), inhibits enzymes in the glycolysis pathway, and uncouples the membrane potential in mitochondria (Newton et al. 2005; Phan and Marquis 2006). The toxicity to different prokaryotic species is thus far from trivial to predict. Basing the hazard estimation of TCS on only a few selected species will likely result in highly biased results that might not be representative of natural bacterial communities.

Previous studies have investigated the effect of TCS on freshwater or estuarine bacterial communities (Drury et al. 2013; Johnson et al. 2009; Lawrence et al. 2009; Lubarsky et al. 2012; Nietch et al. 2013; Proia et al. 2011; Proia et al. 2013; Ricart et al. 2010). Studies of TCS effects on marine bacterial communities are, however, scarce. Johansson et al. (2014) studied effects of TCS on bacterial carbon utilization in marine periphyton communities, in which TCS did not inhibit the carbon utilization and also did not cause changes in bacterial functional diversity at concentrations of up to 10 µM. Eriksson et al. (2015) also studied effects of TCS on carbon utilization in marine periphyton using flow-through microcosms in which TCS did not cause effects at concentrations of up to 1 µM. These studies, however, focused mainly on gross parameters of bacterial function. They do not provide insights into the impact of TCS on microbial diversity.

Amplicon sequencing, also known as metabarcoding, enables the analysis of bacterial communities by analyzing amplicons of marker regions, such as 16S rRNA genes. In contrast to the cultivation of individual strains or metabolic assays such as bacterial carbon utilization, metabarcoding provides an integrative view of a community, including its structure and its individual members (for example Tan et al. 2015). Today, modern sequencing platforms offer massive sequencing depth, which has tremendously increased the sensitivity of amplicon sequencing and allows to detect less and less abundant taxa. Consequently, amplicon sequencing can identify changes in the composition of a bacterial community that would be undetectable with traditional methods such as of microscopy, various molecular fingerprinting techniques (e.g. Terminal Restriction Fragment Length Polymorphism and Denaturing Gradient Gel Electrophoresis), or metabolic assays. There are several examples where metabarcoding has been used to pin-point effects in microbial communities caused by exposure to toxicants (e.g. Chariton et al. 2014; Eriksson et al. 2009; Pascault et al. 2014).

In this study we used 16S rDNA amplicon sequencing to investigate the ecotoxicological effects of TCS on marine periphyton communities that were established under selection pressure from different concentrations of TCS in a flow-through microcosm experiment. The study was implemented to provide information on the impacts of TCS on community composition and diversity in these communities, in order to improve the mechanistic basis for the risk assessment of TCS in marine ecosystems.

## Material and methods

### Flow-through microcosm experiment

A flow-through experiment was performed at the Sven Lovén Centre for Marine Sciences, Kristineberg on the west coast of Sweden from the 26st of September until the 14th of October 2012. The setup of the microcosm system, the operation and implementation of the TCS exposure and the periphyton colonization, as well as the details about the chemical analyses of TCS, the responses of various endpoints (photosynthesis, pigment content, and carbon utilization), are reported in Eriksson et al. (2015). In short, seawater, with its indigenous microbiota, was continuously pumped into 20 L glass aquaria from 1.5 meters depth in the Gullmar fjord. To prevent larger organisms from entering the microcosms, the seawater was filtered through a nylon net with a 1 mm mesh. Periphyton communities colonized and grew on 10.8 cm^2^ (1.4 × 7.7 cm) glass slides mounted vertically in polyethylene holders. Prior to periphyton establishment, the discs were boiled for 10 min in concentrated nitric acid, rinsed in de-ionized water, and rinsed again in 70% ethanol. The seawater flow rate in the microcosms was 220 mL min^−1^ giving a mean residence time of approximately 90 min. TCS solutions were made in de-ionized water by adding TCS dissolved in acetone. These TCS solutions, containing 1 permille acetone (v/v), were pumped into the system at a dilution factor of 119 times, creating constant TCS nominal exposures of 0, 0.316, 1, 3.16, 10, 31.6, 100, 316, and 1000 nM. The same amount of de-ionized water, with the same amount of acetone, but without TCS was pumped into the control microcosms. The study thus had a concentration-response experimental design based on 13 replicates sampled from seven different concentrations of TCS (Table 1). Although some variations were found between the nominal and analyzed TCS concentrations, the nominal concentrations were close to the analyzed concentrations (Eriksson et al. 2015). Therefore, the nominal exposure concentrations are used throughout this paper. Full information of the nominal and analyzed TCS exposure concentrations is given in Eriksson et al. (2015).

**Table 1:** Read and OTU count statistics for 16S amplicons from exposed and unexposed microcosms

Exposure concentration (nM)	Number of replicates (*n*)	Average (*n* > 1) or total (*n* = 1) read count per sample (standard deviation)	Average (*n* > 1) or total (*n* = 1) OTU count per sample (standard deviation)
0	4	15,514 (4525)	844 (63)
0.316	1	13,919	789
1	1	22,658	1262
3.16	2	24,286 (15888)	1102 (402)
10	1	45,545	1141
31.6	2	40,628 (1826)	685 (70)
316	2	19,926 (284)	727 (29)

### Periphyton sampling and DNA extraction

Periphyton biofilms were scraped off with a scalpel from 17 glass slides (183 cm^2^) per microcosm into filter-sterilized water from the respective microcosm. The biofilm material was pelleted by centrifugation at 15,000 g for 8 min, snap-frozen in liquid nitrogen, and stored at −80 °C. DNA extraction was performed using the FastDNA spin kit for soil (MP Biomedicals, Santa Ana, USA) due to the high extraction yield obtained with this kit (Corcoll et al. 2017). DNA extraction was done following the protocol of the manufacturer. Extracted DNA was quantified by fluorescence with the PicoGreen assay (Quant-iT PicoGreen, Invitrogen). The integrity of the extracted DNA was assessed with a 2200 TapeStation instrument (Agilent Technologies, Santa Clara, USA), and contamination by proteins and carbohydrates was quantified as 260/280 nm and 260/230 nm absorbance ratios, respectively, using a NanoDrop 2000 instrument (Thermo Scientific, Wilmington, USA).

### PCR, library preparation, and sequencing

Amplicon 16S rDNA sequences were obtained through a two-step PCR approach as previously described (Sinclair et al. 2015) with some modifications. In short, each sample was first amplified in duplicates using primers targeting the variable 16S regions V3 and V4, equipped with parts of the Thruplex Illumina sequencing adapter. The forward primer: ACACTCTTTCCCTACACGACGCTCTTCCGATCT-NNNN-CCTACGGGNGGCWGCAG and reverse primer: AGACGTGTGCTCTTCCGATCT-GACTACHVGGGTATCTAATCC (Andersson et al. 2010) were used. Duplicates were pooled after purification using the Agencourt AMPure XP system (Beckman Coulter) as recommended by the manufacturer. The pooled duplicates were used as templates in a second PCR step using primers equipped with a 7-base index in the Illumina sequencing adapters for multiplexing. The forward primer

AATGATACGGCGACCACCGAGATCTACAC-[i5 index]-ACACTCTTTCCCTACACGACG and reverse primer

CAAGCAGAAGACGGCATACGAGAT-[i7 index]- GTGACTGGAGTTCAGACGTGTGCTCTTCCGATCT were used to obtain amplicons with complete Thruplex adapters for Illumina sequencing. After sample purification using the Agencourt AMPure XP kit, and quantification by fluorescence with the PicoGreen assay (Quant-iT PicoGReen, Invitrogen), samples were pooled in equimolar amounts. The pooled samples were sequenced at the SciLifeLab SNP/SEQ next generation sequencing facility (Stockholm, Sweden) using Illumina MiSeq with a 2 × 300 bp chemistry following the protocols of the manufacturer.

### Bioinformatics and statistics

The raw sequence data were analyzed with a pipeline for de-multiplexing and sequence-pair assembly implemented in Python (Sinclair et al. 2015). PANDAseq (Masella et al. 2012) was used to assemble the overlapping paired ends (using default settings). Quality filtering removed any sequences with missing primers or unassigned base pairs (Sinclair et al. 2015). Sequences were then clustered into operational taxonomic units (OTUs) based on a 3% dissimilarity clustering with UPARSE, and singleton OTUs were removed (Edgar 2013). Taxonomic annotation was performed using CREST (Lanzen et al. 2012) and the SilvaMod database provided by the online resource SILVA (Quast et al. 2012). The raw sequence data were deposited at NCBI under the BioProject accession number PRJNA320539, and with the SRA Experiment accession numbers SRX1744264–SRX1744266, SRX1744269–SRX1744273 and SRX1744275–SRX1744279.

The Bray-Curtis distance, richness, and evenness were estimated using data rarified to the lowest sequencing depth (*n* = 11,988). Differentially abundant OTUs were identified using the DESeq2 R package. Two types of analyses were implemented: (i) pair-wise analysis between the untreated controls and the samples that were exposed to 3.16, 31.6, and 316 nM TCS, and (ii) regression analysis between OTU counts and TCS concentration using all 13 samples. The resulting p values were adjusted for multiple testing according to Benjamini-Hochbergs false discovery rate (FDR). OTUs with an FDR of less than 0.05 were considered statistically significant. In the pair-wise analysis, overrepresented taxa among the significant OTUs (FDR < 0.05) were tested using Fishers’ exact test at the phylum, class, order, and family levels. Venn diagrams were used to describe the overlap of the significantly different OTUs between concentrations. All analyses were performed in the statistical language R version 3.4 (R Development Core Team 2008).

## Results and discussion

### Results from next-generation sequencing

Sequencing using the Illumina platform resulted in 313,855 16S reads, with an average of 24,143 reads per microcosm (Table 1). The sequence reads from all microcosms were clustered into 1,789 OTUs, with an average of 892 OTUs per sample. The number of OTUs from each treatment is presented in Table 1. Taxonomic annotation of the OTUs revealed a high diversity with 31 prokaryote phyla present in all microcosms (Supplementary Table 1). The phyla *Proteobacteria* and *Bacteroidetes* dominated the communities and constituted 51 and 29% of the OTUs, respectively. These phyla also contained the highest richness with 654 and 449 OTUs, respectively (Supplementary Table 1).

### TCS effects on community composition

**Fig. 1:**
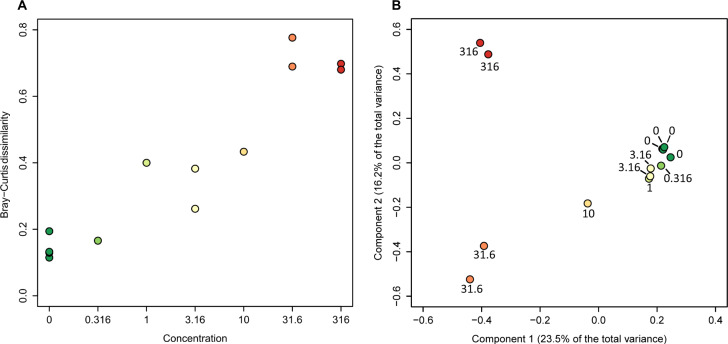
Effects of triclosan on the species composition of marine biofilms. **a** Bray–Curtis similarity of the 16S OTU composition plotted against TCS concentration. **b** Principal components analysis based on Bray–Curtis similarity indices. All concentrations in nM. (Note that two control replicates are similar enough to almost completely overlap.)

TCS exposure clearly changed the OTU distribution of exposed biofilms. The Bray-Curtis dissimilarity between control and exposed communities increased monotonically with increasing TCS concentrations (Fig. 1a). Significant increases in the Bray-Curtis dissimilarity were already observed after an exposure to 1 and 3.16 nM TCS (average difference of 0.21 between the treatments and controls, *p* = 0.0279, Welch test). This pattern is confirmed in the Principal Component Analysis (PCA) (Fig. 1b). Moreover, both the OTU richness and evenness of the communities were significantly reduced at 31.6 and 316 nM (Supplementary Figs. 1 and 2).

**Fig. 2:**
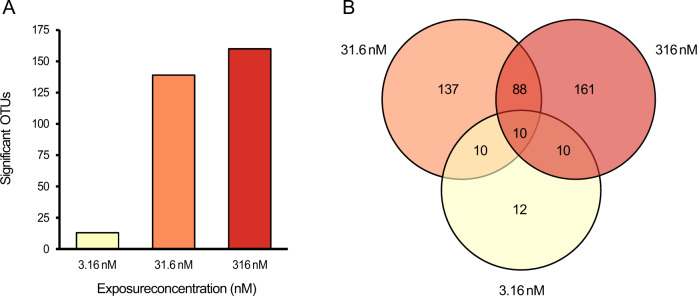
Number of 16S OTUs affected by triclosan exposure. **a** Number of OTUs with significantly different relative abundances, in comparison to unexposed control communities. **b** Number of co-occurring OTUs with significantly different relative abundances, in comparison to unexposed control communities

The relative abundance of a total of 164 OTUs was significantly affected (FDR < 0.05) by an exposure to 3.16, 31.6 or 316 nM TCS (Supplementary Table 3). The number of significantly affected OTUs increased with TCS concentration (Fig. 2a). 10 of the 12 OTUs whose abundance was significantly changed by an exposure to 3.16 nM TCS were also significantly affected at higher exposure levels (Fig. 2b). The abundance of 88 OTUs was significantly affected at both 31.6 and 316 nM, but 29 and 53 OTUs showed such difference only in the 31.6 and 316 nM treatments, respectively, giving these treatments a distinct community profile. The number of OTUs with a significant increased abundance in the treatments compared to the controls were 2, 46, and 70 for 3.16, 31.6, and 316 nM, respectively. The corresponding numbers for OTUs with significant decreased abundance in these treatments compared to the controls were 10, 91, and 91, respectively. We also performed a regression analysis in order to identify OTUs whose abundance correlated with TCS exposure. In total 171 OTUs were found to be significantly correlating with TCS exposure (FDR < 0.05), of which 83 increased and 88 decreased with increasing TCS concentration (Supplementary Table 2).

### Members of the Candidate division *Parcubacteria* are sensitive to triclosan

**Fig. 3:**
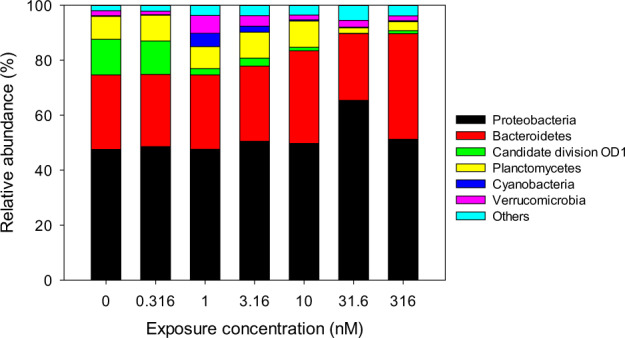
Average relative abundance of the six most abundant bacterial phyla in relation to triclosan exposure

TCS effects were visible already at the phylum level, where OTUs of the Candidate division *Parcubacteria* decreased substantially at a concentration as low as 1 nM (Fig. 3). Fishers’ exact test confirmed that the phylum Candidate division *Parcubacteria* is indeed particularly sensitive, with the abundance of 28% of its taxa showing a significant negative correlation with TCS concentrations (*p* = 4.0 × 10^−6^, Table 2). Also in the pairwise comparison between the exposed and the control communities, the Candidate division *Parcubacteria* was identified as being sensitive, with the abundance of 4.9, 28, and 22% of its taxa being significantly reduced after exposure to 3.16 nM (*p* = 0.029, Table 3), 31.6 nM (*p* = 7.4 × 10^−5^), and 316 nM (*p* = 0.0027) TCS, respectively. The Candidate division *Parcubacteria*, also called OD1, is a diverse group of bacteria, suggested to constitute a superphylum (Solden et al. 2016). Its members have small genomes and reduced metabolic capabilities, lacking genes for the biosynthesis of cofactors, nucleotides, amino acids and fatty acids. Furthermore, *Parcubacteria* have previously been suggested to be symbiotic, commensal, or parasitic organisms (Nelson and Stegen 2015). For example, Gong and co-authors found that the bacterium *Candidatus Sonnebornia yantaiens* was endosymbiotic in the algae *Chlorella*, which in turn was endosymbiontic in the ciliate *Paramecium bursaria* (Gong et al. 2014). As periphyton biofilms also harbor a high diversity of eukaryotic organisms, these communities may be an excellent habitat for such lifestyles. In addition to the TCS-sensitivity demonstrated in this study, *Parcubacteria* have also been shown to be sensitive to oil contamination in soil (Liao et al. 2015). Conceivably, the streamlined genomes and the reduced metabolic capabilities of these organisms makes them unable to handle the metabolic challenges that toxic exposure might present. It is also possible that their symbiotic or commensal interactions are disturbed when their hosts are exposed to toxic compounds, or that the hosts are eliminated by the exposure.

**Table 2:** Overrepresentation of taxa positively or negatively correlated with TCS concentration

Taxonomic group *(Phyla)* * (Class)* * (Order)* * (Family)*	Number of OTUs in taxa^a^	Significant increased (+) or decreased (−) taxa	Percent increased/decreased taxa (%)	*p* value
*Bacteroidetes*	450		7.1/5.3	0.11/0.90
* Flavobacteria*	172	+	15/4.1	1.8 × 10^−6^/0.94
* Flavobacteriales*	167	+	16/4.2	1.0 × 10^−6^/0.93
* Flavobacteriaceae*	97	+	22/4.1	4.2 × 10^−8^/0.89
*Candidate division Gracilibacteria*	40	+	20/5	0.0016/0.75
*Candidate division Parcubacteria*	47	−	2.1/28	0.94/4.0 × 10^−6^
*Proteobacteria*	654	+/−	7.8/9.2	0.0049/0.00040
* Alphaproteobacteria*	222	−	4.5/19	0.85/1.1 × 10^−12^
* Rhizobiales*	7		0/12	1/0.16
* Rhodobiaceae*	7	−	0/57	1/0.00051
* Rhodobacterales*	53	−	5.7/57	0.61/9.6 × 10^−24^
* Rhodobacteraceae*	50	−	4/60	0.80/8.4 × 10^−25^
* Sphingomonadales*	18	+	28/0	0.0028/1
* Erythrobacteraceae*	9	+	44/0	0.0011/1
* Deltaproteobacteria*	145		5.5/6.9	0.62/0.47
* Bdellovibrionales*	59		8.5/8.5	0.26/0.34
* Bdellovibrionaceae*	12	+	25/0	0.029/1
* Gammaproteobacteria*	237	+	13/2.1	3.8 × 10^−6^/1
* Alteromonadales*	63		11/0	0.069/1
* Alteromonadaceae*	40	+	15/0	0.025/1
* Oceanospirillales*	34	+	35/0	1.5 × 10^−7^/1
* Oceanospirillaceae*	19	+	58/0	7.8 × 10^−10^/1
* Thiotrichales*	21	+	19/9.1	0.030/0.52
* Thiotrichaceae*	11	+	36/0	0.0026/1

^a^The number of OTUs of lower taxonomic levels are included in the number of OTUs of higher taxonomic levels. The OTUs for which lower taxonomic levels couldn’t be assigned is included for higher taxonomic levels.

**Table 3:** Overrepresentation of taxa differentially abundant at 3.16 nM TCS compared to controls

Taxonomic group *(Phyla)* * (Class)* * (Order)* * (Family)*	Number of OTUs in taxa^a^	Significant increased (+) or decreased (−) taxa	Percent increased/decreased taxa (%)	*p* value
*Actinobacteria*	46		2.2/0	0.055/1
* Acidimicrobiia*	25	+	4.0/0	0.030/1
* Acidimicrobiales*	25	+	4.0/0	0.030/1
Candidate division *Parcubacteria*	41	−	0/4.9	1/0.029
*Proteobacteria*	599	−	0/1.5	1/0.0027
* Alphaproteobacteria*	204	−	0/3.9	1/5.7 × 10^−6^
* Rhizobiales*	32	−	0/13	1/3.51 × 10^−5^
* Rhodobiaceae*	7	−	0/57	1/3.72 × 10^−8^
* Rhodobacterales*	51	−	0/7.8	1/0.00023
* Rhodobacteraceae*	48	−	0/8.3	1/1.8 × 10^−4^
*Verrucomicrobia*	95		1.1/0	0.11/1
* Verrucomicrobiae*	64		1.6/0	0.076/1
* Verrucomicrobiales*	61		1.6/0	0.073/1
* Rubritaleaceae*	26	+	3.8/0	0.031/1

^a^The number of OTUs of lower taxonomic levels are included in the number of OTUs of higher taxonomic levels. The OTUs for which lower taxonomic levels couldn’t be assigned is included for higher taxonomic levels.

### Proteobacteria can be highly sensitive as well as tolerant to triclosan

In the dominant phyla *Proteobacteria* approximately the same number of taxa were positively and negatively correlated to TCS concentrations (7.8 and 9.2% respectively, Table 2). However, clear patterns in differential TCS sensitivity became evident at lower taxonomic levels, where 19% of the OTUs belonging to *Alphaproteobacteria* were negatively correlated with TCS exposure (*p* = 1.1 × 10^−12^, Table 2). Further down in the alphaproteobacterial taxonomy, 57 and 60% of the OTUs belonging to the order *Rhodobacterales* and the family *Rhodobacteraceae*, respectively, were negatively correlated to TCS exposure (*p* = 9.6 × 10^−24^ and *p* = 8.4 × 10^−25^, respectively, Table 2). The abundance of 8.3% of the OTUs from the family *Rhodobacteraceae* was significantly reduced, already at a TCS concentration of 3.16 nM, (1.8 × 10^−4^, Table 3). As Wagner-Döbler and Biebl (2006) showed, the family *Rhodobacteraceae* harbors the *genus Roseobacter*, whose members may constitute up to 25% of the bacterial community in marine coastal environments. In several studies (Doghri et al. 2015; Michael et al. 2016; Sanli et al. 2015), *Roseobacter* have been shown to be important members of marine biofilms, an observation supported by our study. According to Luo and Moran (2014), members of *Roseobacter* can use a large number of metabolic pathways, including anoxygenic phototrophy, denitrification, methylotrophy, and sulfur oxidation. The genus *Roseobacter* has thus been indicated as an important contributor to the cycling of nutrients in coastal marine environments. The results from our study also reveals that other TCS-sensitive *Alphaproteobacteria* include the order *Rhizobiales* and the family *Rhodobiaceae*. A full 57% of the taxa in the family *Rhodobiaceae* was negatively correlated with TCS exposure (p = 0.00051, Table 2), and the same percentage was underrepresented at 3.16 nM TCS (*p* = 3.72 × 10^−8^, Table 3). *Rhizobiales* are well-known for their nitrogen fixation in symbiosis with legume plants, but as Sanli et al. (2015) showed this order has been detected in the marine biofilms before.

*Alphaproteobacteria* also comprise TCS-tolerant taxa. Of the OTUs in the order *Sphingomonadales* and the family *Erythrobacteraceae*, 28 and 44%, respectively, were positively correlated with TCS exposure (*p* = 0.0028 and 0.0011, respectively, Table 2). Bacterial groups in the family *Erythrobacteraceae*, such as *Erythrobacter*, are non-motile, obligate aerobes and are frequently found in coastal environments. Furthermore, these groups are known to be facultative photoheterotrophs and perform anoxygenic photosynthesis (Koblížek et al. 2003). Yurkov et al. (1996) observed that some *Erythrobacter* displayed resistance to the reactive oxygen species (ROS)-generating compound tellurite, and TCS is well known for its ROS-mediated toxic effects in various organisms (e.g. Li et al. 2018; Pan et al. 2018). We therefore hypothesize that TCS exposure selects for *Erythrobacteraceae* because of their superior ability to handle TCS-induced oxidative stress. Our analysis shows that at least for *Alphaproteobacteria*, the class level is too high to analyze differential TCS sensitivity, as the families of *Rhodobacteraceae* and *Rhodobiaceae* were sensitive but the family *Erythrobacteraceae* was tolerant.

Several *Gammaproteobacteria* were favored by TCS as 13% of its OTUs were positively correlated with TCS exposure (*p* = 3.8 × 10^−6^, Table 2) and 7.4 and 15% of its OTUs showed significantly higher abundances at 31.6 and 316 nM, respectively, compared to unexposed controls (Supplementary Table 2). However, the gammaproteobacterial families *Alteromonadaceae*, *Oceanospirillaceae*, and *Thiotrichaceae* were particularly tolerant, as 15%, 58%, and 36%, respectively, of their corresponding OTUs increased with increasing TCS concentrations (*p* = 0.069, 1.5 × 10^−7^ and 0.0026, Table 2). These results were confirmed in the pairwise comparisons. At 31.6 nM, the abundance of 46 and 40% of the OTUs in *Oceanospirillaceae* and *Thiotrichaceae* were significantly increased (Supplementary Figure 3), and at 316 nM the abundance of 61, 36, and 22% of the OTUs in *Alteromonadaceae*, *Oceanospirillaceae*, and *Thiotrichaceae* were significantly increased. These taxonomic groups were favored only at higher concentrations of TCS (Supplementary Table 2, Supplementary Figure 3). Although *Pseudomonas aeruginosa* belongs to *Pseudomonadales*, i.e. a different gammaproteobacterial order, it is worth noting that *P. aeruginosa* is intrinsically resistant to TCS. This resistance is believed to originate from efflux pumps, but Zhu et al. (2010) showed that *P. aeruginosa* carries a TCS-resistant enoyl-ACP reductase isoenzyme, encoded by the *fabV* gene, which results in a 2000-fold increase of the Minimum Inhibitory Concentration (MIC) of TCS. It is, however, currently not known to what extent other *Gammaproteobacteria* carry a TCS-resistant *fabV* gene. As reviewed by Carey and McNamara (2015), other enoyl-ACP reductase isoenzymes, encoded by the *fabK* and *fabL* genes, can also result in TCS resistance. Furthermore, a combination of resistance mechanisms was induced in the biofilm-forming *Gammaproteobacteria Salmonella enterica serovar Typhimurium* upon TCS exposure, including upregulation of the genes *fabI*, *micF*, *acrAB*, *bcsA*, and *bcsE*. This resulted in increased production of TCS target sites, reduced influx, increased efflux, and increased production of exopolysaccharides (Tabak et al. 2007). Whether these resistance mechanisms are used by periphyton-inhabiting *Gammaproteobacteria* remains to be clarified, but the results presented here supports that *Gammaproteobacteria* in marine biofilms can be tolerant to TCS.

*Deltaproteobacteria* were less abundant than *Alphaproteobacteria* and *Gammaproteobacteria* (Table 2). Similar to the pattern observed in *Alphaproteobacteria*, approximately the same number of taxa in *Deltaproteobacteria* was positively and negatively correlated with TCS exposure. The deltaproteobacterial family *Bdellovibrionaceae* was clearly favored by TCS, where 25% of its OTUs displayed a significant positive correlation to TCS exposure (*p* = 0.029, Table 2). Still, a significant over-representation of taxa only occurred at the highest exposure of 316 nM (Supplementary Figure 3). *Bdellovibrionaceae* predates on other bacteria and was previously thought not to occur in marine waters. However, Kandel et al. (2014) found this family in saline (20 ppt) aquaculture systems, and even showed that *Bdellovibrionaceae* was more abundant in biofilms than in the planktonic phase. Our results thus confirm that *Bdellovibrionaceae* are indeed present in naturally occurring marine biofilms. It seems reasonable to assume that since *Bdellovibrionaceae* predates on other bacteria, this taxon should thrive in biofilms due to the high bacterial density in this habitat. Muller et al. (2011) showed that *Bdellovibrionaceae* has unique membrane lipid structures, but whether this characteristic renders them tolerant to the inhibition of fatty acid synthesis from TCS remains to be clarified.

### Triclosan also affects *Bacteroidetes*, Candidate division *Gracilibacteria*, *Verrucomicrobia*, and *Actinobacteria*

Other examples of bacterial groups clearly favored by TCS were found within the phylum *Bacteroidetes*. The order of *Flavobacteriales* and the family *Flavobacteriaceae* were both significantly overrepresented at 316 nM and having a positive correlation with TCS exposure (*p* = 1.0 × 10^−6^ and *p* = 4.2 × 10^−8^, respectively, Table 2). Many periphytic bacteria are known to degrade alginate and other carbohydrates produced by algae (Klindworth et al. 2014; Zozaya-Valdes et al. 2015). Interestingly, Klindworth et al. (2014) noted that *Flavobacteriaceae* species were the major algal polymer degraders in a diatom bloom, whereas the *Rhodobacteraceae* species exhibited less specialized substrate spectra. If TCS indeed causes mortality in diatom-dominated biofilms, as suggested by the TCS-tolerance pattern of periphytic algae (Eriksson et al. 2015), the fact that *Flavobacteriaceae* are being favored and *Rhodobacteraceae* are being reduced by TCS exposure could be explained by the different substrate spectra of those two groups.

The phylum Candidate division *Gracilibacteria* responded in a similar pattern as *Flavobacteria*, with 20% of their OTUs increasing significantly with TCS concentration (p = 0.0016, Table 2) and only the highest exposure of 316 nM giving a significant over-representation compared to controls. Wrighton et al. (2012) assembled genomes of the Candidate divisions *Gracilibacteria* and *Parcubacteria* from an acetate-amended aquifer and concluded that these organisms have small genomes, are strictly anaerobic, and drive pathways for anoxic carbon, hydrogen, and sulfur cycling similar to those in *Archaea*. In terms of sensitivity to TCS, however, the Candidate divisions *Gracilibacteria* and *Parcubacteria* are not similar, since *Parcubacteria* was clearly TCS sensitive whereas *Gracilibacteria* was tolerant. Hence, small genomes and reduced metabolic capabilities do not seem to determine TCS sensitivity *per se*. The Candidate divisions *Gracilibacteria* and *Parcubacteria* might occupy different ecological niches, and/or have different ecological interactions that are affected by TCS exposure.

A non-monotonic concentration-response pattern, with significant over-representation at 3.16 nM but not at higher exposure levels, was observed for some taxa, for example the family *Rubritaleaceae* in *Verrucomicrobia* (Table 3) and the class *Acidimicrobiia* and the order *Acidimicrobiales* in *Actinobacteria* (Supplementary Figure 4). It is possible that ecological interactions changed at intermediate TCS concentrations, favoring these taxa. For example, *Verrucomicrobia* can be symbionts with ciliates (Vannini et al. 2003) and algae (Ferrero et al. 2012), and *Actinobacteria* can be closely associated with marine sponges (Seipke et al. 2012) and marine macroalgae (Hollants et al. 2013), habitats that are similar to periphyton biofilms. If eukaryotic species symbiontic to *Verrucomicrobia*, or associated with *Actinobacteria*, were favored at intermediate TCS concentrations, these bacterial taxa might increase as well.

### TCS effects on bacterial communities in marine and freshwater ecosystems

The effects of TCS on the composition of natural bacterial communities have been investigated for both freshwater and marine communities. In the freshwater environment, gel-based methods for separating DNA amplicons and FISH have been used, and TCS concentrations of 10 nM (Johnson et al. 2009), 70 nM (Lubarsky et al. 2012), 35 nM (Lawrence et al. 2009), and 6.2 nM (Lawrence et al. 2015) have been shown to change the composition of freshwater communities. In addition, Drury et al. (2013) used 16S amplicon sequencing to study effects of TCS on freshwater sediment communities in artificial streams. These authors found the taxa *Sphingobacteria*, *Betaproteobacteria*, *Deltaproteobacteria*, and *Bacteroidia* to be TCS sensitive, whereas *Anaerolineae* and *Cyanobacteria* were identified as being resistant. In our study with marine biofilms we similarly found some *Sphingobacteria* and some *Deltaproteobacteria* to be TCS sensitive (Table 2), whereas the class *Betaproteobacteria* was not identified as being TCS sensitive.

In marine biofilms, Dobretsov et al. (2007) used T-RFLP and fluorescent *in situ* hybridization (FISH) and found that the overall bacterial density and community composition of 16S in a marine biofilm was affected at a high TCS concentration of 1000 nM, but that the taxa *Alphaproteobacteria* and *Gammaproteobacteria* were affected already at 10 nM. In the present study, we identified Alphaproteobacterial taxa at lower taxonomic levels to be TCS-sensitive (Tables 2 and 3). However, in contradiction to Dobretsov et al. (2007), we found Gammaproteobacterial taxa to be tolerant to TCS (Table 2). The concentrations in which TCS effects were observed in the current study (1–3.16 nM) are lower than those of the studies on freshwater communities cited above. It should be underlined that these changes consisted of changes in the relative OTU composition at lower taxonomic levels. Such changes could be missed if techniques are used that are recording effects at high taxonomic levels or if community-level parameters such as bacterial productivity are used. For example, Eriksson et al. (2015) used Biolog Ecoplates to study TCS effects on bacterial carbon utilization using the same samples from which also the material for the amplicon sequencing efforts of the current study was sourced, and no clear effects of TCS were detected. This is most likely a consequence of the functional redundancy of the carbon utilization of the different taxa, due to which subtle shifts in community composition go unnoticed.

Furthermore, it is important to note that we employed an experimental system with a flow-through setting that continuously brings in new bacteria from the environment. This implies that communities were exposed to TCS during the entire lifecycle of the biofilm, including the colonization phase. TCS effects on the early life stages of a biofilm will then be amplified during the course of its succession. It is therefore likely that the experimental design, in combination with amplicon sequencing, facilitated the identification of significant TCS effects at comparatively low effect levels and concentrations. In particular, the employed experimental strategy allowed us to identify bacterial species, in an ecologically realistic setting, as either particularly TCS-sensitive or –tolerant. Moreover, the concentration-response experimental design containing 13 replicates over seven concentrations was used in order to maximize the sensitivity to identify taxa that change in relative abundance with increasing TCS concentrations. Note however, that a drawback of this design is the reduced statistical power in individual concentrations. However, the vast majority of the effects reported in this study were present in multiple concentrations, strongly suggesting that they were not false positives.

## Conclusions

We identified clear changes in community composition at 10 nM TCS, but effects on specific taxa were seen already at 1–3.16 nM. Our results show that Candidate division *Parcubacteria* and *Alphaproteobacteria* (primarily *Rhodobacteraceae* and *Rhodobiaceae*) are particularly sensitive to TCS while *Gammaproteobacteria* (primarily *Alteromonadaceae*, *Oceanospirillaceae*, and *Thiotrichaceae*), *Flavobacteria* (primarily *Flavobacteriaceae*), the Candidate division *Gracilibacteria*, the deltaproteobacterial family *Bdellovibrionaceae*, and the alphaproteobacterial family *Erythrobacteraceae* are more tolerant to TCS exposure. The results show that TCS affects marine microbial communities at low nanomolar concentrations, which are close to those found in the marine environment (Pintado-Herrera et al. 2014; Remberger et al. 2002). Environmental risk assessments of TCS, such as the recent evaluation published by Guo and Iwata (2017), should therefore include toxicity of triclosan to environmental bacteria and their natural communities.

### Supplementary data

The raw sequence data are deposited at NCBI under the BioProject accession number PRJNA320539, and with the SRA Experiment accession numbers SRX1744264–SRX1744266, SRX1744269–SRX1744273 and SRX1744275–SRX1744279.

## Supplementary material

Supplementary material

Supplementary Table 3
