# Supplementary material for: Triclosan changes community composition and selects for specific bacterial taxa in marine periphyton biofilms in low nanomolar concentrations
Source: Ecotoxicology. 2020 Jul 13;29(7):1083–94. doi: 10.1007/s10646-020-02246-9 (PMC7427700; doi:10.1007/s10646-020-02246-9)
Supplement: Supplementary file 1 — Supplementary material [file 10646_2020_2246_MOESM1_ESM.docx]

**SUPPLEMENTARY MATERIAL**

Article title: Triclosan changes community composition and selects for specific bacterial taxa in marine periphyton biofilms in low nanomolar concentrations

Journal Name:

Author names: Eriksson Karl Martin^a1^*, Sanli Kemal^b1^, Nilsson Rickard Henrik^b^, Eiler Alexander^c^, Corcoll Natalia^b^, Johansson Carl Henrik^b^, Backhaus Thomas^b^, Blanck Hans^b^ and Kristiansson Erik^d^

^a^ Chalmers University of Technology, Department of Mechanics and Maritime Sciences, Gothenburg, Sweden

^b^ University of Gothenburg, Department of Biological and Environmental Sciences, Gothenburg, Sweden

^c^ Uppsala University, Department of Ecology and Genetics, Uppsala, Sweden

^d^ Chalmers University of Technology, Department of Mathematical Sciences, Gothenburg, Sweden

*Corresponding author: K. Martin Eriksson

E-mail address: [martin.eriksson@chalmers.se](mailto:martin.eriksson@chalmers.se)

^1^These authors contributed equally to this work.

Supplementary Table 1. Total 16S OTU abundance and richness of the detected phyla from all microcosms.

| **Phyla** | **Abundance** | **OTU richness** |
| --- | --- | --- |
| *Proteobacteria* | 166098 | 654 |
| *Bacteroidetes* | 90767 | 450 |
| *Planctomycetes* | 20652 | 248 |
| Candidate division *Parcubacteria* | 12620 | 47 |
| *Verrucomicrobia* | 8533 | 100 |
| *Cyanobacteria* | 3312 | 46 |
| Candidate division *Gracilibacteria* | 2979 | 40 |
| *Actinobacteria* | 2633 | 52 |
| Candidate division SR1 | 2412 | 14 |
| *Chlamydiae* | 1225 | 40 |
| *Firmicutes* | 879 | 10 |
| *Chloroflexi* | 588 | 19 |
| *Lentisphaerae* | 241 | 11 |
| *Deinococcus-Thermus* | 192 | 4 |
| *Acidobacteria* | 167 | 11 |
| *Fusobacteria* | 94 | 2 |
| *Candidate division TM7* | 68 | 7 |
| *Gemmatimonadetes* | 47 | 5 |
| *Fibrobacteres* | 44 | 1 |
| Candidate division WS3 | 29 | 3 |
| *Chlorobi* | 26 | 2 |
| *Armatimonadetes* | 25 | 1 |
| Candidate division BRC1 | 24 | 5 |
| Candidate division TM6 | 23 | 3 |
| Candidate division WS6 | 19 | 2 |
| WCHB1-60 | 12 | 1 |
| *Nitrospirae* | 6 | 2 |
| *Spirochaetes* | 6 | 2 |
| Candidate Division OP8 | 2 | 1 |
| Candidate division OP3 | 2 | 1 |
|  |  |  |

Supplementary Table 2. Taxonomic annotation of OTUs with a significant positive/negative correlations to TCS concentration. Note that the number of OTUs at lower taxonomic levels (e.g. *Flavobacteriaceae*) are included in the number of OTUs at the higher taxonomic levels (e.g. *Bacteroidetes*). Hence, some of the OTUs with positive correlation (in total 83 OTUs) and the some of the OTUs with negative correlation (in total 88 OTUs), are counted several times in the column to the right.

| Taxonomic annotation | Number of OTUs in taxa | Number of OTUs with significant positive/negative correlations | | Percent OTUs with significant positive/negative correlations | |  |
| --- | --- | --- | --- | --- | --- | --- |
| *Actinobacteria/Acidimicrobiia/Acidimicrobiales* | 27 | | 0 / 1 | | 0 / 3.7 | |
| *Bacteroidetes* | 450 | | 25 / 18 | | 5.5 / 4.0 | |
| *Cytophagia* | 60 | | 0 / 1 | | 0 / 1.7 | |
| *Flavobacteria/Flavobacteriales* | 166 | | 20 / 5 | | 12 / 3.0 | |
| *Cryomorphaceae* | 31 | | 3 / 1 | | 9.7 /3.2 | |
| *Flavobacteriaceae* | 97 | | 17 / 3 | | 18 / 3.1 | |
| Unknown | 29 | | 0 / 1 | | 0 / 3.4 | |
| *Sphingobacteria/Sphingobacteriales* | 164 | | 5 / 8 | | 3.0 / 4.9 | |
| *Chitinophagaceae* | 18 | | 0 / 1 | | 0 / 5.6 | |
| *Saprospiraceae* | 55 | | 3 / 3 | | 5.5 /5.5 | |
| Unknown | 83 | | 2 / 4 | | 2.4 / 4.8 | |
| Candidate division *Gracilibacteria* | 40 | | 8 / 2 | | 20 / 5 | |
| Candidate division *Parcubacteria* | 47 | | 1 / 10 | | 2.1 / 21 | |
| *Chlamydiae* | 40 | | 1 / 0 | | 2.5 / 0 | |
| *Cyanobacteria* | 46 | | 2 / 0 | | 4.3 / 0 | |
| *Deinococcus-Thermus* | 4 | | 1 / 0 | | 25 / 0 | |
| *Planctomycetes* | 248 | | 3 / 8 | | 1.2 / 3.2 | |
| *Phycisphaerae/Phycisphaerales* | 55 | | 0 / 5 | | 0 / 9.0 | |
| *Phycisphaeraceae* | 24 | | 0 / 2 | | 0 / 8.3 | |
| Unknown | 31 | | 0 / 3 | | 0 / 9.7 | |
| *Planctomycetacia/Planctomycetales/Planctomycetaceae* | 73 | | 0 / 1 | | 0 / 1.4 | |
| Unknown | 19 | | 3 / 2 | | 16 / 11 | |
| *Proteobacteria* | 654 | | 42 / 47 | | 6.4 / 7.2 | |
| *Alphaproteobacteria* | 222 | | 6 / 37 | | 2.7 / 17 | |
| *Rhizobiales/Rhodobiaceae* | 7 | | 0 / 3 | | 0 / 43 | |
| *Rhodobacterales/Rhodobacteraceae* | 50 | | 2 / 25 | | 4.0 / 50 | |
| *Rickettsiales* | 26 | | 0 / 2 | | 0 / 7.7 | |
| *Sphingomonadales* | 18 | | 3 / 0 | | 17 / 0 | |
| *Erythrobacteraceae* | 9 | | 2 / 0 | | 22 / 0 | |
| *Sphingomonadaceae* | 8 | | 1 / 0 | | 13 / 0 | |
| Unknown | 19 | | 1 / 4 | | 5.2 / 21 | |
| *Deltaproteobacteria* | 145 | | 8 / 7 | | 5.6 / 4.8 | |
| *Bdellovibrionales* | 59 | | 5 / 4 | | 8.5 / 6.8 | |
| *Bdellovibrionaceae* | 12 | | 3 / 0 | | 25 / 0 | |
| Unknown | 33 | | 2 / 4 | | 6.0 / 12 | |
| *Desulfuromonadales* | 19 | | 1 / 0 | | 5.3 / 0 | |
| *Myxococcales* | 22 | | 2 / 1 | | 9.1 /4.5 | |
| *Nannocystaceae* | 11 | | 1 / 0 | | 9.1 / 0 | |
| Unknown | 6 | | 1 / 1 | | 17 / 17 | |
| Unknown | 18 | | 0 / 2 | | 0 / 11 | |
| *Gammaproteobacteria* | 237 | | 27 / 2 | | 11 / 0.84 | |
| *Alteromonadales* | 63 | | 6 / 0 | | 9.5 / 0 | |
| *Alteromonadaceae* | 40 | | 5 / 0 | | 13 / 0 | |
| Unknown | 8 | | 1 / 0 | | 13 / 0 | |
| *Chromatiales* | 11 | | 2 / 0 | | 18 / 0 | |
| *Ectothiorhodospiraceae* | 1 | | 1 / 0 | | 100 / 0 | |
| *Granulosicoccaceae* | 7 | | 1 / 0 | | 14 / 0 | |
| *Oceanospirillales/Oceanospirillaceae* | 19 | | 9 / 0 | | 47 / 0 | |
| *Thiotrichales/Thiotrichaceae* | 11 | | 4 / 1 | | 36 / 9.1 | |
| Unknown | 7 | | 6 / 1 | | 86 / 14 | |
| Unknown | 9 | | 0 / 1 | | 0 / 11 | |
| *Verrucomicrobia* | 100 | | 0 / 1 | | 0 / 1 | |

Supplementary Figure 1. Richness, measured as the unique number of OTUs, for each concentration of TCS. For replicated concentrations, the bar represents the standard error. The richness in 31.6 and 316 nM was significantly decreased compared to the controls (p=0.0236 and p=0.0169 respectively).

Supplementary Figure 2. Evenness, measured using Pielous’ index, for each concentration of TCS. For replicated concentrations, the bar represents the standard error. The richness in 31.6 and 316 nM was significantly decreased compared to the controls (p=8.18×10^-5^ and p=0.0299 respectively).


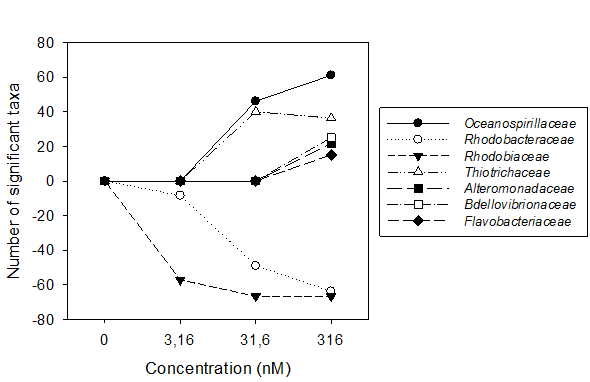


Supplementary Figure 3. Percentage of significant over- and under-represented taxa in families from pairwise comparisons between controls and exposure treatments. Only families containing three or more taxa are included. Significance determined as adjusted p-value < 0.05 in Fisher’s exact test.


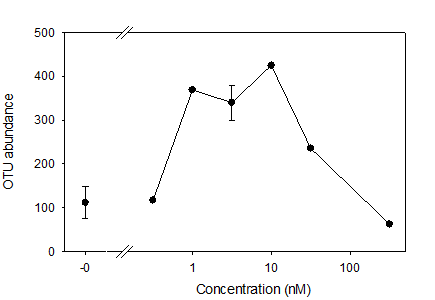


Supplementary Figure 4. Abundance of *Actinobacteria* OTUs at different concentrations of TCS. Error bars denote standards error of the mean.
